# Genome Sequence of a Carbapenem-Resistant Strain of *Ralstonia mannitolilytica*

**DOI:** 10.1128/genomeA.00405-15

**Published:** 2015-05-07

**Authors:** Masato Suzuki, Hisaaki Nishio, Kohsuke Asagoe, Kaneyuki Kida, Satowa Suzuki, Mari Matsui, Keigo Shibayama

**Affiliations:** a Department of Bacteriology II, National Institute of Infectious Diseases, Musashimurayama, Tokyo, Japan; b Department of Clinical Laboratory, Shiga Medical Center for Adults, Moriyama, Shiga, Japan; c Department of Hematology and Oncology, Shiga Medical Center for Adults, Moriyama, Shiga, Japan; d Department of Clinical Laboratory Medicine, Otsu Red Cross Hospital, Otsu, Shiga, Japan

## Abstract

*Ralstonia mannitolilytica*, a Gram-negative aerobic bacterium, is an opportunistic human pathogen that is becoming more common in cases of nosocomial infections. We report for the first time the whole-genome sequence analysis of *R. mannitolilytica* strain MRY14-0246, which carries the intrinsic OXA-443/OXA-22-like and OXA-444/OXA-60-like β-lactamase genes and is resistant to meropenem.

## GENOME ANNOUNCEMENT

*Ralstonia mannitolilytica*, a Gram-negative aerobic bacterium belonging to the genus *Ralstonia*, is prevalent in water supplies and is becoming more common in cases of nosocomial infections (1). *R. mannitolilytica* is closely related to *Ralstonia pickettii* and had previously been named “*Pseudomonas thomasii*” and *R. pickettii* biovar 3/“*thomasii*” (1, 2). In 2001, *R. mannitolilytica* was classified as a novel species in the genus *Ralstonia*, based on 16S rRNA gene sequence analysis (2). *R. mannitolilytica* is known as an opportunistic human pathogen, possibly associated with cystic fibrosis (3), and the clinical isolates frequently exhibit resistance to imipenem (4). However, little is known about drug resistance and virulence phenotypes of *R. mannitolilytica* because its genetic basis is uncertain.

In this report, we announce the first draft genome sequence of *R. mannitolilytica*. *R. mannitolilytica* strain MRY14-0246 was recovered from a patient’s urine in a medical institution in Japan and was resistant to meropenem, according to the MIC determined using the Vitek2 system and an Etest (bioMérieux) and applying the recommended breakpoints described by the CLSI (5). Whole-genome shotgun sequencing of strain MRY14-0246 was performed using the Illumina HiSeq 2500 pyrosequencing platform (500- to 750-bp insert size). Paired-end reads (2 × 150 bp) were assembled *de novo* using CLC Genomics Workbench version 7.5.1 (Qiagen). The draft genome sequence of strain MRY14-0246 consisted of 48 contigs, yielding total sequences of 4,671,011 bp with *N*_50_ contig sizes of 328,267 bp. The mean G+C content was 65.8%. A total of 4,357 coding DNA sequences were annotated by the RAST server version 2.0 (http://rast.nmpdr.org). The 16S rRNA gene sequence of strain MRY14-0246 almost matched that of the *R. mannitolilytica* type strain LMG 6866^T^ (GenBank accession number AJ270258) (99.8%). Strain MRY14-0246 carried two novel class D β-lactamase gene variants, which we named *bla*_OXA-443_ and *bla*_OXA-444_ (accession numbers LC030178 and LC030179, respectively).

The *bla*_OXA-443_ and *bla*_OXA-444_ genes in strain MRY14-0246 are encoded in contigs 8_1 and 10_1 (accession numbers BBUP01000016 and BBUP01000018), respectively, both of which are parts of the chromosome with no transposable element, suggesting that these oxacillinase genes are intrinsically species-specific in *R. mannitolilytica*. *R. pickettii* produces two resident oxacillinases named OXA-22 and OXA-60 (6, 7). Unlike OXA-22, which is a narrow-spectrum oxacillinase, OXA-60 is an extended-spectrum oxacillinase with carbapenem-hydrolyzing properties (6). The OXA-443 and OXA-444 proteins exhibit close similarities to OXA-22 and OXA-60 (86.0% and 90.3% amino acid identities), respectively, suggesting that OXA-444/OXA-60-like β-lactamase could contribute to carbapenem resistance in strain MRY14-0246.

Bacterial pathogens frequently use protein secretion systems to interact with their hosts. The type III secretion system (T3SS) and type VI secretion system (T6SS) are known as major virulence factors of the plant pathogen *Ralstonia solanacearum* (8, 9). Strain MRY14-0246 does not contain the T3SS gene cluster, whereas the strain contains the T6SS gene cluster and two *hcp* and four *vgrG* translocator genes. T6SS delivers effectors into neighboring organisms, including bacteria and hosts, leading to cytotoxicity and cell death of targets (10). Hence, T6SS could be an important virulence determinant in *R. mannitolilytica*. A more detailed report of strain MRY14-0246 will be included in a future publication.

### Nucleotide sequence accession numbers.

The whole-genome shotgun projects of *R. mannitolilytica* strain MRY14-0246 have been deposited at DDBJ/EMBL/GenBank under the accession number BBUP00000000. The version described in this paper is the first version, BBUP00000000.1.
